# Endovascular Bail Out for a Failed Transcatheter Aortic Valve Implantation in High Surgical Risk Patient

**DOI:** 10.1155/2021/8858935

**Published:** 2021-03-09

**Authors:** Ilaria Peluttiero, Alessandro Robaldo, Luigi Leotta, Claudio Novali

**Affiliations:** Department of Vascular and Endovascular Surgery, S.Croce e Carle Hospital, Cuneo, Italy

## Abstract

The endovascular treatment for acute type A dissection (ATAD) represents an alternative and emerging option in selected high surgical risk patients. We report a successful total endovascular ATAD repair occurred intraoperatively during transcatheter aortic valve implantation (TAVI) placement in 82 years old female, not fit for surgery in emergency setting. The presentation, the diagnostic evaluation, and the technique are discussed. This case would support the feasibility and efficacy of the stent graft technology to treat ATADs after evaluation of clinical, anatomical, and radiological parameters.

## 1. Introduction

The surgical repair of the ascending aorta represents a technical challenge particularly in patients with adverse anatomy and extensive comorbidities. Although nowadays, the endovascular repair represents the gold standard for the descending thoracic aortic pathologies, and its role in treating ascending aortic lesions remains debatable. In addition to the fact that endografts designed to treat the aortic ascending segment are not currently available, its use is restricted by some anatomical and hemodynamic considerations associated with the risk of procedure-related cardiac complications [1]. In the literature, some reports showed the feasibility of the endovascular ascending solution for acute/chronic aortic dissections, aneurysms, intramural hematoma, and traumatic ruptures with an acceptable rate of complications [2]. The aim of our report is to show the potential and safe use of the endovascular treatment for an acute type A dissection after transcatheter aortic valve implantation (TAVI) in emergency setting.

## 2. Case Presentation

**Figure 1:**
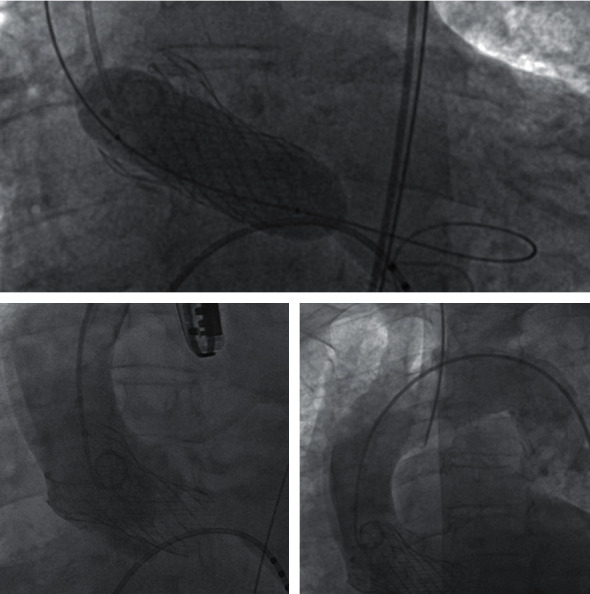
TAVI procedure and ascending aorta dissection.

**Figure 2:**
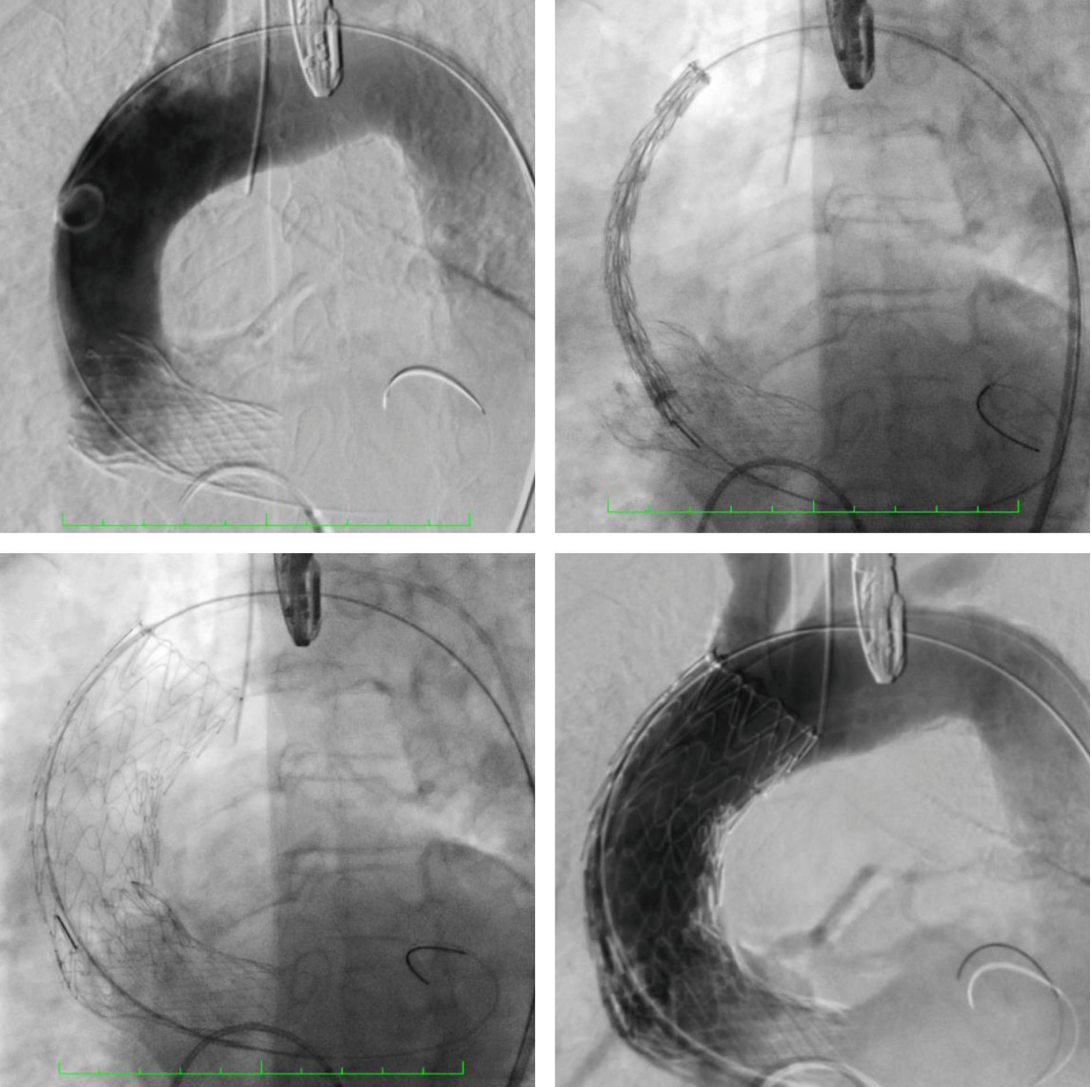
Endograft deployment and final result.

An 82-year-old white female, with a history of hypertension, obesity, chronic heart failure, and severe chronic obstructive pulmonary disease was referred to the department of cardiology for a severe aortic valve stenosis with symptom of angina. A TAVI implantation (Medtronic Evolut R 29) was scheduled. After ballooning with valvuloplasty catheter (Valver 25 mm), the procedure was intraoperatively complicated by the dissection of the ascending aorta after the valve implantation (Figure 1). Due to the high surgical risk and the fact that patient's anatomy met the inclusion criteria, a total endografting solution was chosen to cover the main entry tear in an emerging setting. A thoracic stent-graft (Gore, C-TAG: TGU31-31-10) was brought to the ascending aorta through the right femoral artery over an exchange length 0.035′′ Amplatz super-stiff guidewire. Aortography from the sinus of Valsalva was used to mark the reference points after the localization of the entry tear, the orifice of the coronary arteries, the leaflets of the implanted valve, and the branches of the aortic arch. Before the deployment under rapid ventricular pacing and transesophageal echocardiography control, the “nose” cone of the device passed through the implanted valve, into the left ventricle to allow the appropriate proximal landing zone of the stent-graft. Final angiography revealed excellent covering of the main entry tear and patency both of the coronary ostia and the innominate artery without any complications (Figure 2). The postoperative course was uneventful. The patient was discharged in good condition after 7 days with a 6-month dual antiplatelet therapy. Follow-up at 3 months, including cardiological and neurological observation, demonstrated no complications. Currently, the patient refused to undergo a contrast-enhanced multidetector computed tomography angiography (MDCTA).

## 3. Discussion

Open surgical repair remains the gold standard option for ATAD. However, some authors report that up to 25% of those patients are too high risk and deemed inoperable [3]. These patients may benefit from a less invasive alternative with thoracic endovascular aortic repair, but its role for treating ascending aortic pathology is less well known. Currently, different types of thoracic stent grafts were used in an offlabel fashion to perform this technique [4]. Nevertheless, a high percentage (30%) of patients with an ascending aortic disease was unsuitable for TEVAR due to presence of unfavorable anatomical characteristics [1]. Currently, some selected clinical conditions include pseudoaneurysm, postsurgery bleeding, residual dissection and ATAD. Potential percentage of patients fit for ascending TEVAR ranges from 31.5% to 36.2% due to the average increase in the mid ascending aortic diameter of 32%, the ascending/aortic arch forces, and the length mismatch between the inner and outer curves [5]. Furthermore, the planning should consider that the use of standard thoracic stent-graft could be precluded by the fact that the average ascending aorta length ranges from 7 to 9 cm, and the covered area is limited both proximally by the coronary arteries ostia and distally by the supra-aortic trunk.

The procedure limitations included the presence of both a mechanical aortic valve and the surgical grafts originating from the middle segment of the vessel as a previous aortocoronary bypass. In our case, we met all the anatomical requirements including a proper femoral access. This is the most used access, but the transapical, the axillary, and the carotid approaches can be an alternative solution. In particular, we want to highlight the fact that the previous placement of the TAVI allowed us to extend the proximal landing zone and to measure accurately the stent-graft size, although the surgical strategy and the type of graft were chosen in an emergency setting.

The endovascular repair of the ascending aorta could be an alternative and safe treatment in selected high-risk patients with acute type A dissection who are unfit for surgical repair. The literature review reports that both technical and clinical outcomes are promising but they will require further assessment to prove the safety and efficacy of the technique [2].
